# Did the COVID-19 Lockdown Reduce Smoking Rate in Adolescents?

**DOI:** 10.3390/ijerph20010139

**Published:** 2022-12-22

**Authors:** Seunghyup Lee, Mingee Choi, Dahyun Kim, Jaeyong Shin, Junghyun Kim

**Affiliations:** 1 Department of Chemical and Biomolecular Engineering, Yonsei University, 50, Yonsei-ro, Seodaemun-gu, Seoul 03722, Republic of Korea; 2 Interdisciplinary Graduate Program in Social Welfare Policy, Yonsei Graduate School, Yonsei University, 50, Yonsei-ro, Seodaemun-gu, Seoul 03722, Republic of Korea; 3 Department of Statistics, Sungshin Women’s University, 2, Bomun-ro 34da-gil, Seongbuk-gu, Seoul 02844, Republic of Korea; 4 Department of Preventive Medicine, Yonsei University College of Medicine, 50-1, Yonsei-ro, Seodaemun-gu, Seoul 03722, Republic of Korea; 5 Institute for Innovation in Digital Healthcare, Yonsei University Health System, Seoul 03722, Republic of Korea

**Keywords:** COVID-19, electronic nicotine delivery devices, prevention, adolescents, smoking

## Abstract

This study examined the temporal trend of smoking use and the prevalent differences in the use of different types of cigarettes for Korean adolescents before and during the COVID-19 pandemic. In Korea, all use of e-cigarettes, including regular cigarettes, is considered smoking. Since adolescents are susceptible to peer influences in risky behaviors including smoking, social distancing could affect the smoking behaviors of youth under these unusual circumstances during the pandemic. In this study, we analyzed the Korea Youth Risk Behavior Web-based Survey (KYRBW) data collected from 2018 to 2021 to examine the association between smoking status and other covariates during the COVID-19 pandemic. As a result, it was confirmed that the influence of second-hand smoke on the smoking rate decreased before and after COVID-19, which is interpreted as a result of the social distancing policy caused by the pandemic.

## 1. Introduction

Smoking among the young population remains a leading public health concern worldwide. Adolescent smoking damages pulmonary functions by changing the peripheral airway and leads to dysfunction. Structural changes and immunological responses to the respiratory system owing to smoking increases the risk of bacterial and viral respiratory infections during smoking in teenagers. In addition, the study found that high-density lipoprotein-cholesterol, which is classified as ‘good cholesterol’, decreased in adolescents who had been smoking for less than 2 years [1]. Nicotine use among youth damages brain development by disrupting cognitive functions, increasing the risk of lifelong tobacco addiction, and may also increase the risk of future addiction to other drugs [2,3].

According to the Juvenile Protection Act in Korea, the age of 19 years is the minimum legal age for purchasing and smoking tobacco products. However, many adolescents in Korea can access and consume cigarettes illegally. According to the Korea Youth Risk Behavior Web-based Survey (KYRBWS) in 2018, approximately 0.1 million Korean middle and high school students reported current (past 30-day) use of tobacco products. Adolescents’ cigarette smoking prevalences were 9.8% and 3.0% for high school and middle school students, respectively, in 2018. The total prevalence of adolescent cigarette smoking has decreased from 12.1% in 2010, 9.2% in 2014, and 6.3% in 2016 to 6.7% in 2018. Despite the downward trend in adolescent smoking in Korea, the diversification of tobacco products, including electronic cigarettes (e-cigarettes) and heat-not-burn tobacco, and recent marketing strategies of the tobacco industries through social media have significantly impacted imitation, curiosity, and positive image formation of tobacco smoking among adolescents [4,5,6,7,8,9,10].

Previous studies identified that adolescents are susceptible to peer influences and engage in risky behaviors, including smoking [11,12,13]. The likelihood of smoking increases when they are socializing with peers who smoke, are pro-smoking, or are exposed to people who are smoking [14,15]. In addition, tobacco use is initiated and established primarily during youth and young adulthood; 44% of adults initiated smoking under the age of 18, 28% of teen smokers in Korea reported having tried their first cigarette in the 9th grade, and Korean adolescents start smoking at a mean age of 12.7 years, which is lower than the international mean age of 13–15 years [16,17]. Finally, smoking in adolescence leads to nicotine addiction and increases the probability of continuing smoking later in life. Therefore, it is important to prevent tobacco exposure among adolescents by implementing policies that restrict adolescent smoking. Since the influence of peers is great in encouraging youth smoking, it can be inferred that youth smoking has decreased due to the social distancing policy or school blockade policy caused by the COVID-19 pandemic.

As of 31 May 2022, more than 18.1 million people have been infected and approximately 24,200 have died from coronavirus disease 2019 (COVID-19) in Korea. The 1st, 2nd, and 3rd waves of the COVID-19 outbreak peaked on 20 January 2020, 28 July 2020, and 3 November 2021, respectively. To limit the outbreaks, the government implemented public health interventions, including mandatory indoor and outdoor face masking, restricted public gatherings, and working from home without lockdown. For youth, in-person class attendance was strictly limited to two-thirds or capped at one-third of the student body depending on the COVID-19 situation in Korea. Social distancing could affect the smoking behaviors of youth under these unusual circumstances during the pandemic.

Scrutinizing how the pandemic impacted cigarette use behaviors is important to plan for cigarette control in youth. There have been no population-based studies assessing and characterizing access and use of cigarette smoking among adolescents before and during the pandemic era in Korea. Monitoring such patterns and their impact may provide age-specific public health insights for the prevention of adolescent cigarette adoption. Thus, we decided to evaluate whether the COVID-19 pandemic reduced the smoking rates in adolescents.

## 2. Materials and Methods

### 2.1. Study Design

Our study population included teenagers aged 12–18 years who participated in the KYRBWS from 2018 to 2021. A total of 227,139 survey data were used in this study, of which 51.8% were men and 48.2% were women. In terms of age, a similar proportion of responses were shown. A total of 117,343 and 109,796 students were in the ‘Before COVID-19 (2018–2019)’ and ‘During COVID-19 (2020–2021)’, respectively. The KYRBWS is a representative survey by the Korean government and is conducted due to the participation of teenagers in classrooms with computers every year; the survey includes general health status questionnaires. Any missing values in the questionnaire were excluded from the analysis, which excluded 17,810 data (approximately 10%). The KYRBWS was reviewed by the Korea Centers for Disease Control and Prevention’s Institutional Bioethics Committee with government-approved statistics (Approval No. 11758) based on the National Health Promotion Act. All data used in this study are publicly available on the KYRBWS website.

The primary outcomes were the changes in the smoking status of different types of cigarettes during COVID-19. Furthermore, changes in the smoking status were analyzed according to sex, housing income, educational background, parents’ education level, and secondhand smoking.

### 2.2. Statistical Analysis

Categorical variables were compared between groups using the chi-square test. Multiple logistic regression analysis with adjustment for covariates was performed to examine the association between the smoking status and the pandemic. The 2018–2019 response was set before COVID-19, and the 2020–2021 response was set during COVID-19. All statistical analyses were performed using SAS version 9.4. *p* < 0.05 was considered statistically significant. Since the KYRBWS data included multi-level sampling, layering, and clustering, we analyzed it by applying weights. Responses with logical errors and outliers were processed as missing values.

## 3. Results

### 3.1. General Information on Study Observation

A total of 227,139 students were reviewed. As shown in Table 1, 51.89% and 48.11% of students were male and female, respectively. Among the middle and high school students who participated in the survey, 16.28%, 16.24%, and 16.15% of students were in the 7th, 8th, and 9th grades, respectively, and they were classified as ‘middle school students’ in Korea. In addition, 16.38%, 16.97%, and 17.98% of students were in the 10th, 11th, and 12th grades, respectively, and they were classified as ‘high school students. ’Korean students who smoke cigarettes and e-cigarettes account for 5.59% and 2.99% of all students, respectively. The overall smoking rate was 6.16%. In addition, secondhand smoking rate, income level, academic level, and parents’ educational level were used as variables.

### 3.2. Overall Results of Current Smoking Rates

As shown in Table 2, 7.19% of Korean students were smoking in the period ‘Before COVID-19’, and it slightly increased to 7.32%. After the first COVID-19 case, the current smoking rate in Korean students in 2020 was 4.85%, which was significantly lower than in 2019. Subsequently, it slightly increased to 5.19% [Supplementary material].

### 3.3. Prevalence of Cigarette Smoking before and during COVID-19

Table 3 is the result of analyzing the influencing factors related to youth cigarette smoking before and during COVID-19 through multiple logistic analyses. Both before COVID-19 and during COVID-19, the smoking rate was significantly lower for female students than for male students, and the smoking rate was higher when the house income was low. The change in the influencing factors due to the pandemic was secondhand smoke at home and school, which was shown as an influencing factor before COVID-19 but was not statistically significant during COVID-19.

### 3.4. Prevalence of E-Cigarette Smoking before and during COVID-19

Table 4 is the result of analyzing the influencing factors related to youth e-cigarette smoking before and during COVID-19 through multiple logistic analyses. Similar to the general cigarette result, both before COVID-19 and during COVID-19, the smoking rate was significantly lower for female students than for male students. However, in the case of e-cigarettes, the smoking rate was higher when the house income was high. The change in the influencing factors due to the pandemic was secondhand smoke at home, which was shown as an influencing factor before COVID-19 but was not statistically significant during COVID-19.

## 4. Discussion

This study provides initial insight into the overall impact of the COVID-19 pandemic on adolescent smokers in South Korea. To the best of our knowledge, this is the first study that addresses changes in the use of specific tobacco products before and during the pandemic in Korea. According to the Korea Food Forum, the liquid e-cigarette was first released in 2003, and the Heat-Not-Burn E-cigarette was first sold in 2017. Previous studies have shown that the use of e-cigarettes increases attitudes toward smoking [18,19]. The results of this study also show that the smoking rate in 2019 increased compared with 2018 (Table 2). However, the results of the study depict that the quantity of past-30-day tobacco product use, including conventional cigarettes and e-cigarettes, reduced in adolescent smokers after the worldwide outbreak when compared with the levels before COVID-19. The estimated mean reduction in tobacco use was 2.24% from before to during the pandemic. The estimated mean reduction in conventional, e-liquid, and heat-not-burn cigarettes were 2.24%, 0.54%, and 0.05%, respectively. This is in line with the findings of another study that showed that e-cigarette users aged below 20 years in the United States and Canada had changed their vaping behaviors and were likely to report decreased usage, but there were significant differences compared with our study [20,21].

As a result of analyzing the factors influencing the youth smoking rate in this study, the change in the influence of secondhand smoking before and after COVID-19 was confirmed. In the analysis results before COVID-19, it was confirmed that secondhand smoking at home had an effect on the increase in both cigarette and e-cigarette smoking rates but did not have a significant influence during COVID-19. In the case of general cigarettes, secondhand smoking in schools showed the same results. This result can be interpreted as due to the social distancing policy caused by the pandemic.

Social distancing efforts owing to the COVID-19 pandemic had complex effects. Increased time in smoke-free home environments, intermittent visits to schools, and distance from peers who smoke may have led to a decrease in tobacco use among adolescents. In addition, financial constraints may reduce access to tobacco products. Increased health concerns owing to COVID-19 being a respiratory disease in combination with smoking being harmful to the respiratory system have increased concerns among smokers about having severe illness from a coronavirus infection [22]. Such anxieties motivate some smokers to stop smoking [23]. Since the influence of peers is great in encouraging youth smoking, it can be inferred that youth smoking has decreased due to the social distancing policy or school blockade policy caused by the COVID-19 pandemic. Parental oversight is also related to the decrease in smoking [6], and it can be interpreted that youth smoking has decreased due to increased surveillance as the proportion of parents working from home due to the pandemic.

Comparing the changes in the grade when teenagers first smoked among those who are currently smoking, the rate of smoking for the first time in 7th grade increased after COVID-19, which is consistent with previous reports. [Supplementary material] [20] Although it is illegal for Korean youths to buy cigarettes in South Korea, smoking rates in these age groups persist. Therefore, it is necessary to strengthen the online verification of teenagers who buy and smoke cigarettes. Smoking initiation at a younger age is a strong factor for them to smoke more cigarettes per day, be more likely to continue smoking, and are uniquely vulnerable to developing lifelong nicotine addiction, even at low levels of nicotine use [24]. The mean age of smoking initiation for youth in Korea is much younger than that in other countries. Across 204 countries, the mean age at initiation of regular tobacco smoking was 19.2 years. The mean age at initiation ranged from 16.4 years (16.2–16.7) in Denmark to 22.5 years (22.0–23.1) in Togo [25].

Given the rapidly evolving social media landscape, adolescent youth are influenced by social media platforms, and smoking-related media has increased in recent years [26,27,28]. Marketing and depiction of smoking via social media induce curiosity, since often this content targets adolescents with attractive images [7]. Youths develop positive attitudes toward smoking after seeing their peers post smoking-related content on social media [6]. More than 50% of smoking scenes are presented in web-based cartoons, movies, and dramas that Korean students enjoy, and 86% of popular YouTube channels display smoking [29].

Although this study confirms the trends of tobacco use among Korean youth, it has limitations. Since smoking status was self-reported in KYRBWS, there might be response bias. Despite the use of an anonymous online survey method, there may have been an underreporting of smoking. This survey data was collected only from the youth who attended school during 2018–2021. The survey has been used since 2018 because the measurement method of items related to smoking has changed since then and new questions related to smoking have been added at that point. Thus, this cannot be generalizable for all regions, sociodemographic factors, and those who do not attend schools in Korea. Since this is a cross-sectional survey, we can determine the relationship, but not the cause and effect, between smoking and the pandemic. Our research focused on adolescent smokers in Korea. Generalizability to other regions in terms of culture, economic development, and pandemic-related health policy requires further exploration. In the future, it would be meaningful to examine how many current smokers during the COVID-19 pandemic are new smokers and identify the factors affecting the upward trend in smoking after the pandemic.

## 5. Conclusions

The prevalence of cigarette smoking has decreased among adolescents in South Korea. However, smoking will remain an issue for generations to come if tobacco use in adolescents is not substantially reduced. Implementation of age-appropriate and age-tailored interventions are warranted to prevent youth from becoming susceptible to cigarette use and engaging in harmful cigarette use behavior. Surveillance of tobacco use in youth is necessary to understand if the current reduction in smoking among youth during the pandemic is a temporary trend. To end the tobacco epidemic, we must aggressively implement evidence-based strategies to prevent adolescents from initiating smoking and interrupt the constant steady increase in youth smokers.

## Figures and Tables

**Table 1:** General Information of Study Observation.

		Total			Before COVID-19 (2018–2019)		During COVID-19 (2020–2021)		*p* Value
		N	Percent	Std Err	Percent	Std Err	Percent	Std Err	
Age (Mean, SE)		(15.16 ± 0.01)			(15.12 ± 0.02)		(15.21 ± 0.02)		0.0001
Sex	Male	117,058	51.89	0.59	52.02	0.87	51.76	0.80	0.8430
	Female	110,081	48.11	0.59	47.98	0.87	48.24	0.80	
School grade	7th grade	39,606	16.28	0.14	15.21	0.19	17.41	0.20	<0.0001
	8th grade	39,556	16.24	0.13	15.52	0.19	17.00	0.19	
	9th grade	39,427	16.15	0.13	16.41	0.19	15.87	0.18	
	10th grade	35,901	16.38	0.13	16.51	0.18	16.24	0.19	
	11st grade	36,637	16.97	0.14	17.13	0.19	16.81	0.19	
	12nd grade	36,012	17.98	0.15	19.23	0.22	16.67	0.20	
Cigarette	none	214,850	94.41	0.09	93.32	0.14	95.55	0.10	<0.0001
	Over 1/month	12,289	5.59	0.09	6.68	0.14	4.45	0.10	
E-Cigarettes	none	220,683	97.01	0.06	96.71	0.09	97.32	0.07	<0.0001
	Over 1/month	6456	2.99	0.06	3.29	0.09	2.68	0.07	
Total Smoking	none	213,650	93.84	0.10	92.75	0.15	94.98	0.11	<0.0001
	Over 1/month	13,489	6.16	0.10	7.25	0.15	5.02	0.11	
Secondhand smoke in home	none	167,235	74.16	0.13	72.77	0.18	75.63	0.18	<0.0001
	Over 1/month	59,904	25.84	0.13	27.23	0.18	24.37	0.18	
Secondhand smoke in school	none	195,471	85.66	0.19	79.22	0.32	92.43	0.17	<0.0001
	Over 1/month	31,668	14.34	0.19	20.78	0.32	7.57	0.17	
Secondhand smoke in indoor	none	122,552	53.33	0.17	48.06	0.22	58.89	0.24	<0.0001
	Over 1/month	104,587	46.67	0.17	51.94	0.22	41.11	0.24	
House income	High	89,619	40.15	0.20	40.28	0.26	40.01	0.29	<0.0001
	Middle	108,739	47.53	0.15	46.87	0.20	48.24	0.23	
	Low	28,781	12.32	0.10	12.86	0.14	11.75	0.13	
Academic level	High	86,037	37.73	0.15	38.42	0.20	37.00	0.23	<0.0001
	Middle	68,248	30.14	0.10	29.74	0.14	30.57	0.15	
	Low	72,854	32.13	0.13	31.84	0.18	32.43	0.20	
Ease of purchase of cigarettes	No purchase attempt or impossible	218,174	95.86	0.07	95.05	0.11	96.72	0.08	<0.0001
	Buy	8965	4.14	0.07	4.95	0.11	3.28	0.08	
Father education	Under university	130,327	55.84	0.25	54.89	0.36	56.84	0.34	0.0003
	University	96,812	44.16	0.25	45.11	0.36	43.16	0.34	
Mother education	Under university	133,280	57.53	0.24	57.32	0.35	57.75	0.33	0.4083
	University	93,859	42.47	0.24	42.68	0.35	42.25	0.33	
Drinking status	None/month	197,377	86.60	0.12	84.03	0.19	89.30	0.15	<0.0001
	Over 1/month	29,762	13.40	0.12	15.97	0.19	10.70	0.15	

**Table 2:** Overall Results of Current Smoking Rates.

	2018		2019		2020		2021	
	Percent	Std Err	2019	Std Err	2020	Std Err	2021	Std Err
Cigarette	6.68	0.21	6.69	0.19	4.41	0.16	4.48	0.14
Liquid E-cigarette	2.69	0.11	3.16	0.11	1.89	0.08	2.86	0.1
Heat-Not-Burn E-cigarette	-	-	2.62	0.1	1.08	0.06	1.36	0.06
Total Smoking	-	-	7.32	0.2	4.85	0.16	5.19	0.16
Total	7.19	0.22	7.32	0.2	4.85	0.16	5.19	0.16

**Table 3:** Prevalence of Cigarette’s Smoking Before and During COVID-19.

Multiple Logistic Regression		OR		(95% CI)		*p* Value
Before COVID-19 (2018–2019)						
Grade	7th grade	1 [Reference]				<0.0001
	8th grade	2.412	1.931		3.012	
	9th grade	2.569	2.056		3.211	
	10th grade	3.402	2.729		4.242	
	11st grade	3.35	2.686		4.178	
	12nd grade	3.432	2.756		4.275	
Sex	Male	1 [Reference]				<0.0001
	Female	0.435	0.399		0.474	
House income	High	0.938	0.848		1.038	0.0243
	Middle	0.887	0.809		0.971	
	Low	1 [Reference]				
Drinking status	None/month	1 [Reference]				<0.0001
	Over 1/month	6.888	6.378		7.44	
Father education	Under university	1 [Reference]				<0.0001
	University	0.801	0.731		0.877	
Mother education	Under university	1 [Reference]				0.2019
	University	0.942	0.86		1.032	
Academic level	High	0.491	0.451		0.536	<0.0001
	Middle	0.63	0.579		0.686	
	Low	1 [Reference]				
Secondhand smoke in home	None/month	1 [Reference]				0.0004
	Over 1/month	1.143	1.062		1.229	
Secondhand smoke in school	None/month	1 [Reference]				<0.0001
	Over 1/month	1.183	1.093		1.281	
Secondhand smoke in indoor	None/month	1 [Reference]				<0.0001
	Over 1/month	1.451	1.348		1.561	
Ease of purchase of cigarettes	No purchase attempt or impossible	1 [Reference]				<0.0001
	Buy without effort	41.983	38.527		45.749	
**During COVID-19 (2020–2021)**						
Grade	7th grade	1 [Reference]				<0.0001
	8th grade	1.469	1.167		1.85	
	9th grade	2.001	1.616		2.48	
	10th grade	2.646	2.119		3.303	
	11st grade	2.971	2.38		3.707	
	12nd grade	3.46	2.776		4.312	
Sex	Male	1 [Reference]				<0.0001
	Female	0.546	0.496		0.603	
House income	High	0.966	0.855		1.091	0.0003
	Middle	0.823	0.732		0.925	
	Low	1 [Reference]				
Drinking status	None/month	1 [Reference]				<0.0001
	Over 1/month	8.006	7.333		8.741	
Father education	Under university	1 [Reference]				0.3058
	University	0.943	0.843		1.055	
Mother education	Under university	1 [Reference]				0.0006
	University	0.82	0.732		0.918	
Academic level	High	0.412	0.369		0.46	<0.0001
	Middle	0.594	0.533		0.663	
	Low	1 [Reference]				
Secondhand smoke in home	None/month	1 [Reference]				0.7663
	Over 1/month	0.985	0.895		1.086	
Secondhand smoke in school	None/month	1 [Reference]				0.1083
	Over 1/month	1.126	0.974		1.301	
Secondhand smoke in indoor	None/month	1 [Reference]				<0.0001
	Over 1/month	1.516	1.393		1.65	
Ease of purchase of cigarettes	No purchase attempt or impossible	1 [Reference]				<0.0001
	Buy without effort	72.845	64.942		81.709	

**Table 4:** Prevalence of E-Cigarette’s Smoking Before and During COVID-19.

Multiple Logistic Regression		OR		(95% CI)		*p* Value
Before COVID-19 (2018–2019)						
Grade	7th grade	1 [Reference]				<0.0001
	8th grade	3.052	2.211		4.212	
	9th grade	3.085	2.284		4.166	
	10th grade	3.64	2.674		4.956	
	11st grade	3.462	2.551		4.699	
	12nd grade	3.498	2.581		4.743	
Sex	Male	1 [Reference]				<0.0001
	Female	0.361	0.324		0.403	
House income	High	1.068	0.942		1.212	0.0001
	Middle	0.877	0.772		0.996	
	Low	1 [Reference]				
Drinking status	None/month	1 [Reference]				<0.0001
	Over 1/month	5.257	4.75		5.818	
Father education	Under university	1 [Reference]				<0.0001
	University	0.714	0.639		0.797	
Mother education	Under university	1 [Reference]				0.2337
	University	0.937	0.841		1.043	
Academic level	High	0.738	0.664		0.820	<0.0001
	Middle	0.704	0.629		0.788	
	Low	1 [Reference]				
Secondhand smoke in home	None/month	1 [Reference]				<0.0001
	Over 1/month	1.409	1.291		1.537	
Secondhand smoke in school	None/month	1 [Reference]				<0.0001
	Over 1/month	1.517	1.376		1.671	
Secondhand smoke in indoor	None/month	1 [Reference]				<0.0001
	Over 1/month	1.325	1.204		1.458	
Ease of purchase of cigarettes	No purchase attempt or impossible	1 [Reference]				<0.0001
	Buy without effort	16.862	15.290		18.597	
**During COVID-19 (2020–2021)**						
Grade	7th grade	1 [Reference]				<0.0001
	8th grade	1.736	1.353		2.228	
	9th grade	1.916	1.521		2.414	
	10th grade	2.538	2.017		3.194	
	11st grade	2.373	1.886		2.987	
	12nd grade	2.432	1.928		3.068	
Sex	Male	1 [Reference]				<0.0001
	Female	0.604	0.541		0.675	
House income	High	1.136	0.973		1.327	0.0003
	Middle	0.902	0.78		1.043	
	Low	1 [Reference]				
Drinking status	None/month	1 [Reference]				<0.0001
	Over 1/month	5.855	5.261		6.516	
Father education	Under university	1 [Reference]				0.127
	University	0.904	0.794		1.029	
Mother education	Under university	1 [Reference]				0.0001
	University	0.774	0.678		0.883	
Academic level	High	0.681	0.602		0.770	<0.0001
	Middle	0.798	0.703		0.905	
	Low	1 [Reference]				
Secondhand smoke in home	None/month	1 [Reference]				0.0044
	Over 1/month	1.171	1.051		1.306	
Secondhand smoke in school	None/month	1 [Reference]				<0.0001
	Over 1/month	1.808	1.552		2.107	
Secondhand smoke in indoor	None/month	1 [Reference]				<0.0001
	Over 1/month	1.385	1.253		1.531	
Ease of purchase of cigarettes	No purchase attempt or impossible	1 [Reference]				<0.0001
	Buy without effort	25.180	22.350		28.370	

## Data Availability

All data used in this study are publicly available on the KYRBWS website.

## Supplementary Materials

The following supporting information can be downloaded at: https://www.mdpi.com/article/10.3390/ijerph20010139/s1.
