# Supplementary material for: Did the COVID-19 Lockdown Reduce Smoking Rate in Adolescents?
Source: Int J Environ Res Public Health. 2022 Dec 22;20(1):139. doi: 10.3390/ijerph20010139 (PMC9819646; doi:10.3390/ijerph20010139)
Supplement: Supplementary file 1 [file ijerph-20-00139-s001.zip › ijerph-2040142-supplementary.pdf]

Supplementary Figure S1. Current Smoking Rate (2018-2021)

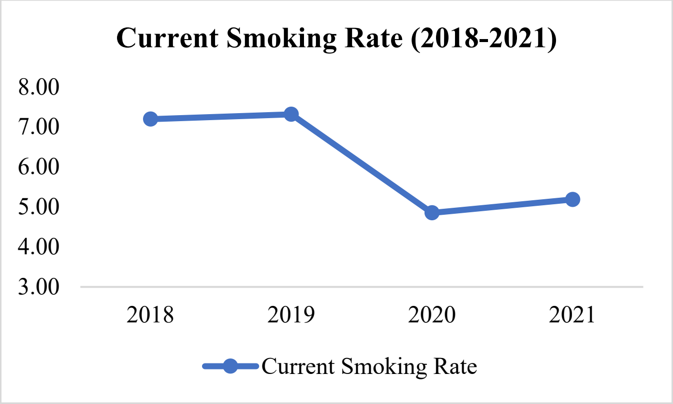

Supplementary Figure S2. Ease of purchase of cigarettes

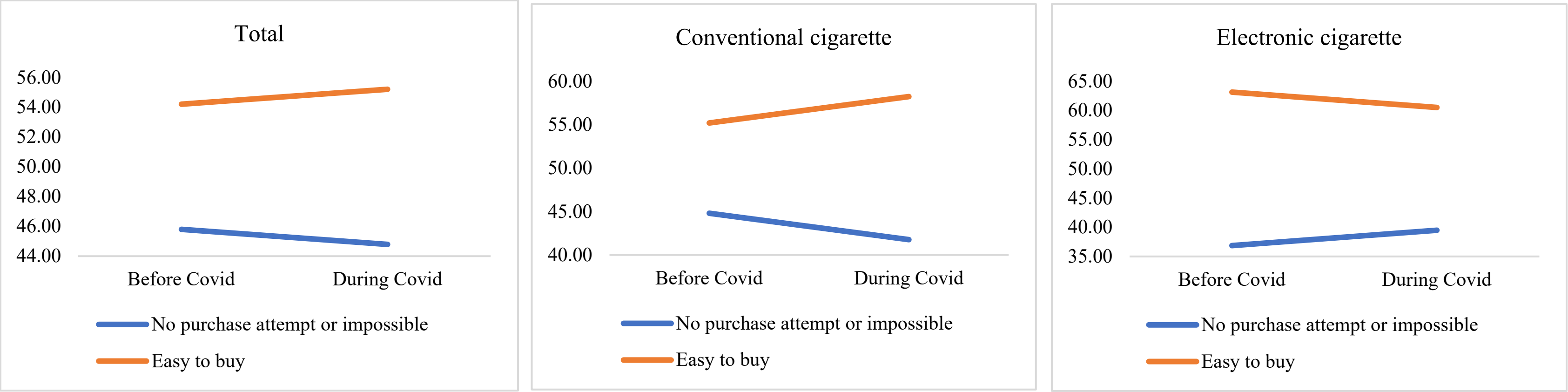

Supplementary Table S1. The grade first started smoking

	Before COVID-19 (2019)		During COVID-19 (2020-2021)		P-value
	Percent	Std Err	Percent	Std Err	
**Grade**					
**Before entering school**	4.34	0.38	2.52	0.25	<.0001
**1st grade**	1.15	0.17	0.38	0.09	
**2nd grade**	0.75	0.14	0.47	0.11	
**3rd grade**	1.87	0.22	0.96	0.16	
**4th grade**	2.34	0.24	1.56	0.17	
**5th grade**	3.49	0.28	3.00	0.23	
**6th grade**	7.64	0.44	6.77	0.36	
**7th grade**	21.19	0.71	26.23	0.64	
**8th grade**	23.75	0.69	27.75	0.67	
**9th grade**	15.16	0.56	14.41	0.51	
**10th grade**	11.92	0.58	10.83	0.48	
**11st grade**	4.57	0.37	3.60	0.24	
**12nd grade**	1.84	0.23	1.53	0.18	
